# The Broad Clinical Spectrum of Epilepsies Associated With Protocadherin 19 Gene Mutation

**DOI:** 10.3389/fneur.2021.780053

**Published:** 2022-01-17

**Authors:** Giovanni Battista Dell'Isola, Valerio Vinti, Antonella Fattorusso, Giorgia Tascini, Elisabetta Mencaroni, Giuseppe Di Cara, Pasquale Striano, Alberto Verrotti

**Affiliations:** ^1^Department of Pediatrics, University of Perugia, Perugia, Italy; ^2^Pediatric Neurology and Muscular Diseases Unit, Istituto di Ricovero e Cura a Carattere Scientifico (IRCCS) “G. Gaslini” Institute, Genoa, Italy; ^3^Department of Neurosciences, Rehabilitation, Ophthalmology, Genetics, Maternal and Child Health, University of Genoa, Genoa, Italy

**Keywords:** PCDH19, epilepsy and mental retardation limited to female (EFMR), GEFS, Dravet syndrome, antiseizure medication (ASM)

## Abstract

Protocadherin 19 (PCDH19) gene is one of the most common genes involved in epilepsy syndromes. According to literature data PCDH19 is among the 6 genes most involved in genetic epilepsies. PCDH19 is located on chromosome Xq22.1 and is involved in neuronal connections and signal transduction. The most frequent clinical expression of PCDH19 mutation is epilepsy and mental retardation limited to female (EFMR) characterized by epileptic and non-epileptic symptoms affecting mainly females. However, the phenotypic spectrum of these mutations is considerably variable from genetic epilepsy with febrile seizure plus to epileptic encephalopathies. The peculiar exclusive involvement of females seems to be caused by a cellular interference in heterozygosity, however, affected mosaic-males have been reported. Seizure types range from focal seizure to generalized tonic-clonic, tonic, atonic, absences, and myoclonic jerks. Treatment of PCDH19-related epilepsy is limited by drug resistance and by the absence of specific treatment indications. However, seizures become less severe with adolescence and some patients may even become seizure-free. Non-epileptic symptoms represent the main disabilities of adult patients with PCDH19 mutation. This review aims to analyze the highly variable phenotypic expression of PCDH19 gene mutation associated with epilepsy.

## Introduction

**Figure 1:**
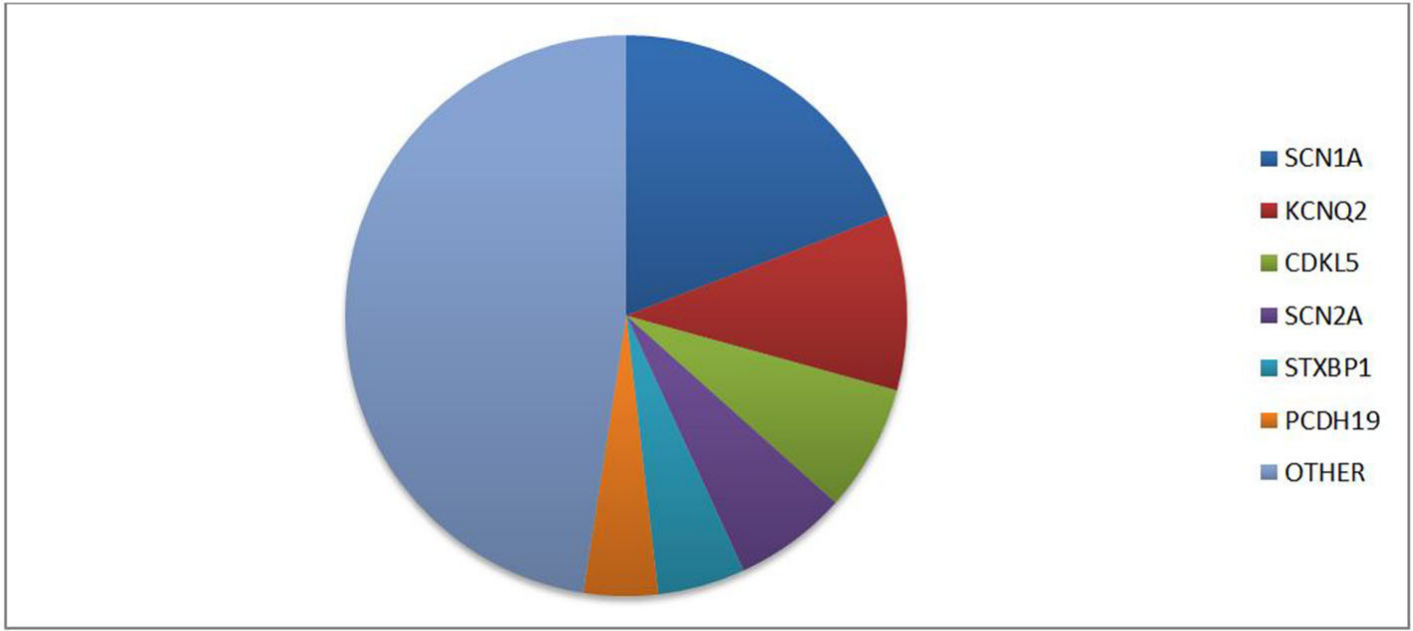
Prevalence of different gene mutations in genetic epilepsies according to literature data [data from (2)].

The latest ILAE classification (1) emphasizes the importance of an etiological classification of epilepsy to improve prognosis and, whether possible, initiate a target therapy. In fact, besides allowing a stratification of risk based on the genotype-phenotype correlation, different patterns of gene mutations could present specific drug-response and lead to target therapy. The main genes associated with epilepsy can be classified based on five different functions: (I) ion transport; (II) cell growth and differentiation; (III) synaptic processes regulation; (IV) transport and metabolism within and between cells of small molecules; and (V) gene transcription and translation (2). One of the most commonly implicated genes in epilepsy is the protocadherin 19 (PCDH19) gene located on chromosome Xq22.1. According to Symonds et al. (2) PCDH19 is among the six genes most involved in genetic epilepsies (Figure 1). PCDH19 gene is expressed in several organs, but primarily in the limbic areas of the nervous system (3). It has a six-exon structure that encodes a transmembrane adhesion molecule of the Cadherin family. The Cadherin superfamily comprises three subgroups of transmembrane cell adhesion molecules: cadherins, protocadherins, and desmosomal cadherins (4). Among these protocadherins represent the main subgroup with approximately 80 members involved in neuronal connections and signal transduction (5, 6). Almost 150 PCDH19 mutations have been described as either familiar clustering or *de novo* (7). Most mutations involve the extracellular protein domains encoded by exon 1 and are typically missense variations (7, 8). However, a few mutations of intracellular domains have also been reported, possibly affecting the intracellular signal pathway. Proper development of neural architecture and neuronal connectivity require efficacious cell-cell interactions and alterations of protocadherins could result in severe disruption in early brain morphogenesis. It is not well known how mutations of PCDH19 lead to the development of epilepsy. However, a role of this gene in the proliferation of neuronal progenitors and the regulation of cell motility during the early stages of neurulation has been proposed (9, 10). Recent *in vitro* studies, conducted with patient-derived induced pluripotent stem cells showed an accelerated differentiation in cells with PCDH19 mutation. It is also observed that increased neurogenesis occurs earlier in PCDH19-mutated culture with an increased neurite length and occurrence of premature neural rosettes. Accelerated neurogenesis is involved with a defect in the cell division plane at the stage of the neural progenitors. Moreover, it is possible that PCDH19 mutations can alter the correct positioning of the mitotic spindle causing a higher number of asymmetric divisions leading to accelerating neural differentiation. An altered equilibrium between symmetric vs. asymmetric cell division may contribute to the pathogenesis of the disease (11). The synaptogenesis role of PCDH19 was confirmed by Mincheva-Tasheva et al. who showed disruption of excitatory synaptic contacts between PCDH19-knock-out and wild-type neurons in “mosaic” neuronal cultures (12). In addition, PCDH19 mutation leads to a decrease in N-cadherin-dependent signaling resulting in an impaired mossy fiber synapse development (13). The interaction between the cytoplasmic domains of PCDH19 with the alpha subunits of the GABAa receptor could alter the excitatory-inhibitory balance underlying epilepsy (14). Besides regulating GABAa receptor surface expression, PCDH19 also regulates channel gating. Indeed, PCDH19-mutated cortical neurons have a spontaneous Ca^2+^ intracellular flux suggesting increase excitability of these cells (15). Another theory suggests PCDH19 involvement in blood-brain barrier (BBB) dysfunction. In fact, the gene is highly expressed in endothelial cells and the main epileptic foci involve the limbic region, close to the periventricular region where BBB is missing. Furthermore, this theory could explain the seizure remission with growth thanks to the maturation of BBB (16). The phenotypic spectrum of PCHD19 mutation is extremely variable involving neurological and psychiatric diseases. Although epilepsy and mental retardation limited to female (EFMR) is the most frequent clinical expression of PCDH19 mutation, other important clinical manifestations are genetic epilepsy with febrile seizure plus (GEFS+) and epileptic encephalopathies. This review aims to analyze the phenotypic expression of PCDH19 gene mutation associated with epilepsy.

## Literature Search

We reviewed the papers (English language only) on PCDH19-related epilepsy through a Literature search on PubMed until August 2021. The terms “PCDH19” and “PCDH19” epilepsy were used in this systematic search. We included case reports and open-label studies. Moreover, we searched for additional articles through a review of the reference lists of published reviews.

## Epilepsy and Mental Retardation Limited to Female

**Figure 2:**
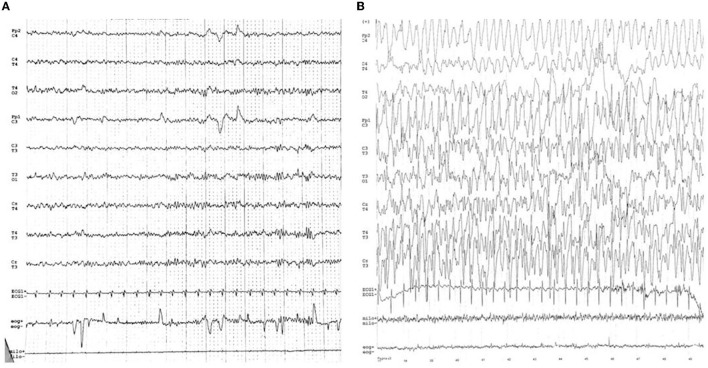
**(A)** Normal inter-ictal EEG. **(B)** Ictal-EEG characterized by frontal theta activity of the left hemisphere followed by sharp waves on the same derivations. Slow waves spread on the frontal derivations of the right hemisphere, followed by diffuse slow waves (except on the right posterior deviations).

Epilepsy and mental retardation limited to female (EFMR) was first described by Juberg and Hellman (17) in 15 related females presenting epilepsy with cognitive impairment. This clinical manifestation was only later related to mutations of the PCDH19 gene (8, 18). Brain MRI is generally normal and there are no peculiar electroencephalographic (EEG) features of PCDH19 mutation. Figure 2 shows an EEG of a female patient with PCDH19 mutation and focal epilepsy. The phenotypic expression of EFMR is characterized by epileptic and non-epileptic symptoms. The hallmark feature especially in the early stages of the disease is cluster focal seizures with the tendency to prolonged episodes poorly responsive to antiepileptic therapy (19). The patient often displays early-onset seizures (6–36 months) generally sensitive to fever (20). Seizure types range from focal seizure to generalized tonic-clonic, tonic, atonic, absences, and myoclonic jerks. The severity of epilepsy is also extremely variable from drug-resistant and progressive forms to self-limiting ones. Seizures become less severe with adolescence while non-epileptic symptoms represent the main disabilities of adult patients with PCDH19 mutation (21). Non-epileptic features of EFMR include intellectual disability (ID) and behavior disturbances occurring in 75.4 and 55.4% of patients, respectively (21). In a study of 195 patients with PCDH19 mutations, only 28.2% had normal cognitive development, while patients with mild, moderate, and severe impairment were 27.2, 22.2, and 17.4%, respectively (7). A delay in the acquisition of language milestones represents a common feature of all patients and the absence of language before seizure onset represents a possible negative prognostic factor for cognitive development (22). Although most patients manifest cognitive delay after 2 years of age, 15% of cases present intellectual disabilities before the onset of epilepsy. Therefore, intellectual impairment is only partially related to epileptic encephalopathy and other genetic and/or environmental factors are among the causes of the phenotypic spectrum. The peculiar exclusive involvement of females seems to be caused by a cellular interference in heterozygosity. According to this theory, the coexistence of mutated cells with wild-type cells causes the neural network alteration at the base of the disease (17, 23). Confirming this hypothesis, while hemizygotic males are asymptomatic carriers, affected mosaic-males have been reported (24). Indeed, postzygotic somatic variants in males would configure a picture overlapping with that of females in heterozygosity. A mutation penetrance of ~80% was estimated for both conditions (7, 25). The small number of affected males makes difficult a phenotypic characterization of these patients. The phenotypic spectrum of mosaic-males resembles that of affected females. In addition, psychiatric comorbidity has also been described in two males with germline mutation, albeit the possible correlation with the mutation is questionable (25). Several studies have looked for a genotype-phenotype correlation without any result. According to a recent study, missense variants seem to be more commonly related to normal cognitive development compared to the loss of function mutations (26). Whole gene deletion appears as well associated with a worsened prognosis. According to Shibata et al. truncating variants located from extracellular domain 5 (EC5) to the cytoplasmic domain present a later seizure onset with less severe intellectual disability compared to missense variants and truncating variants from EC1 to EC4 (27). Cognitive impairment does not appear to be directly associated with seizure severity (22). To date, age at seizure onset and seizure frequency are the only unfavorable prognostic factors associated with cognitive function (7, 26). Indeed, the occurrence of new synapses' processing and changes in the frontal cortex predominantly in the first years of life may justify this correlation (7). In addition, the epileptic expression, also the neuropsychiatric profile is extremely heterogeneous, ranging from mild to severe forms with combination of autistic, attention-deficit/hyperactive, obsessive, or aggressive features (28). Approximately 25% of patients without intellectual disability presents psychiatric comorbidity (7). In addition, there is data that sleep alterations is a common feature in patients with EFMR. Both difficulty in maintaining sleep and in absorption have been reported (28). A more complete analysis of these disorders could be useful especially in light of the correlation between sleep disturbances and worse control of epileptic symptoms. The wide variability in the phenotypic expression of EFMR could be partially explained by X-inactivation in females. However, further studies are needed for a greater knowledge of the impaired biological processes in EFMR and to investigate possible genotype-phenotype correlations.

## Genetic Epilepsy With Febrile Seizures Plus

Febrile seizures (FS) are the most common clinical presentation of GEFS+, followed by febrile seizures plus (FS+). However, absence, myoclonic, atonic, or focal seizures combined with the most frequent phenotypes are also common. Confirming the various spectrum of this disorder, epileptic encephalopathy like Dravet syndrome (DS) and epilepsy with myoclonic-atonic seizures (MAE) are possible manifestations in GEFS+ families. The clinical presentation of PCDH19-related epilepsy often overlaps with that of GEFS+. In both, focal seizures with variable degrees of intellectual disabilities can be part of the phenotypic spectrum. To define a GEFS+ family are necessary two or more individuals with GEFS+ phenotypes including at least one with FS or FS+ (29–31). GEFS+ was initially described as a genetic disorder following an autosomal dominant pattern of inheritance with incomplete penetrance (29, 32, 33). However, some sporadic cases suggested that *de novo* mutation or polygenic pattern could also cause the disease (31, 33–36). With a better knowledge of the clinical presentation, it is now possible to recognize GEFS+ even outside a family context when *de novo* mutations in the GEFS+ gene are found (36). Several genes mutations are described underlying GEFS+ (37–48) including PCDH19 (49, 50). SCN1A is the most commonly involved gene, whose mutations are identified in 19% of affected families (30, 37). There is a known similarity between the phenotypic spectrum of PCDH19 and SCN1A, which includes DS and extends to GEFS+. Recognizing PCDH19 mutations in the context of a GEFS+ presentation can be difficult. However, seizure clusters prevalent in the females should point toward the search for PCDH19 mutations.

## Dravet-Like Syndrome

Dravet syndrome (DS), originally named “severe myoclonic epilepsy of infancy,” represents one of the most severe genetic epilepsy with childhood onset. According to the ILAE classification, the typical DS is defined by febrile and afebrile seizures that occur in the first year of life in an infant with normal development and the subsequent appearance of myoclonus, atypical absences, and focal seizures. Seizures become drug-resistant leading to poor prognosis with motor, cognitive, and psychiatric impairment of affected patients (1, 51). SCN1A is the most frequently involved gene occurring in 70–80% of DS (52, 53). DS-like phenotype is a similar condition due to the occurrence of other gene mutations involved in encephalopathy. In a recent study, mutations in the PCDH19 gene appear to be the underlying cause of DS-like phenotype in 16% of DS negative for SCN1A mutations (54). The variants in the SCN1A and PCDH19 genes show some similarities that link DS to DS-like, but they differ from each other for some peculiarities (55). Due to the unusual X-linked inheritance of PCDH19 gene mutations, DS-like is more common in females. The onset of symptoms is earlier in DS than in DS-like, with an average range of 3–6 and 8–54 months, respectively (56, 57). Fever represents the main triggering seizure factor for both DS and DS-like, however PCDH19 mutations show fewer provocation factors for seizure initiation (55). Clonic and hemiclonic seizures are mainly related to SCN1A mutations that also present a higher prevalence of generalized tonic-clonic seizures and atypical absences with more common status epilepticus. Whereas, seizure types associated with PCDH19 variants are often focal and hypomotor seizures with a higher prevalence of cluster seizures (57). Seizures with affective symptoms and fearful screaming have been described by many authors as a characteristic feature of DS-like (19). DS-like phenotype is less associated with photosensitivity compared to DS. Another relevant difference is the interval for the second seizure occurrence: 10 months for DS-like vs. 2–3 months for DS, probably due to the higher frequency of seizures in the first year of life and the earliest onset in DS patients (20, 57). Interictal EEG may have no abnormalities, however focal or generalized slow wave, sharp and polyspike discharges have been reported (22). DS-like phenotype carrying the PCDH19 pathogenic variants has greater variability in cognitive disability including some cases without intellectual impairment (20, 22, 57). In contrast, patients with DS present a greater degree of cognitive impairment, regardless of variant significance (55). Autism predominantly involves DS-like, occurring in 62.5% of patients with PCDH19 mutations vs. 37.5% of patients with SCN1A mutations (55).

## Treatment of Pcdh19-Related Epilepsy

Treatment of PCDH19-related epilepsy is limited by drug resistance and by the absence of specific treatment indications. These patients usually need polytherapy frequently with poor efficacy due to the natural fluctuating trend of seizures and to the various cluster triggers. The management of drug-refractory patients represents a great challenge for physicians, especially for syndromes with heterogeneous seizure semeiology and course (58). Currently, different drug associations have been tested and none has definitively proven to be superior (Table 1). However, familiar mutations show the same reactivity to antiseizure medication (ASM), therefore the pharmacological choice can be oriented in cases of affected patients in the same family (50). Despite DS, the use of sodium channel blockers such as lamotrigine and carbamazepine in PCDH-19-related epilepsy has shown less seizure exacerbation (22). Bromide and clobazam revealed higher efficacy in reducing seizures after 3 months of treatment with a partial reduction in effectiveness during long-term follow-up (63). Valproate and levetiracetam resulted among the most efficacious antiepileptic drugs with a response rate of 61 and 57%, respectively after 12 months of use (63, 65). The effectiveness of phenytoin is unclear, Higurashi et al. showed a good response, not confirmed by Lotte et al. who reported a high grade of ineffectiveness and seizures worsening after phenytoin administration (60, 63). However, the small sample of patients treated with phenytoin in these studies raises doubts about the reliability of these results. Based on the similarities between DS and PCDH-19 related epilepsy, stiripentol was used in addition to valproate and clobazam in a female patient affected by PCDH19-related resistant epilepsy with a great efficacy (66). Stiripentol was later used, in six patients with PCDH-19 related epilepsy as an add-on to valproate and clobazam with a decrease of seizure frequency by more than 50% (56). It is not clear whether the efficacy obtained after stiripentol introduction was due to the intrinsic effect of the drug or to pharmacokinetic interactions causing an increase of clobazam and valproate blood levels (56, 66). PCDH19 gene mutation can be associated with reduced steroidogenesis. Confirming this hypothesis, Tan et al. identified dysregulated AKR1C1-3 which is involved in the production of allopregnanolone (67). Global decrease of neuroactive steroids such as allopregnanolone, pregnenolone sulfate, 17-OH progesterone, and cortisol could be related to seizures onset in PCDH19 mutation. Thus, restoring steroidogenesis can be a therapeutic goal that may improve the management of this disorder (68). Although corticosteroids can be used to control seizure clusters, lacks a long-term benefit with a high risk of recurrences after interruption (22). Oral corticosteroid prophylaxis during febrile episodes was used in a Japanese study with no recurrence of moderate/severe clusters (57). The ketogenic diet showed a positive response in 50% of patients. Vagus Nerve stimulation was used in only one case with a 75–90% seizure reduction at 3 months, persistent after 1 year (63). A single case report described an improvement in seizure control and development after leucovorin therapy in a patient with low cerebral folate levels (69). In conclusion, similarly to DS, GABAergic drugs are the most effective in the treatment of PCDH19-related epilepsy. In particular, first-line drugs that should be considered are bromide, clobazam, and valproate. Levetiracetam should be considered in patients with highly refractory clusters of seizures. Despite the predominance of focal seizures, carbamazepine does not appear as effective as expected, however sodium channel blockers showed relatively good effectiveness in some patients. Stiripentol may be effective especially in patients with DS-like, however, due to drug resistance, it is often necessary to use it in combination with clobazam and valproate. Aggravations of seizures were reported in connection with sodium channel blockers (58), with topiramate and valproate (60). The increasing number of antiseizure medications in the last decades has led to the development of new successful therapies. Recently, fenfluramine and cannabidiol have proved to be well tolerated and effective in reducing seizures frequency in DS (70, 71). Based on the existence of a similar therapeutic response between DS and PCDH19-related epilepsy, these therapies could be considered in the treatment of the latter patients. Seizure clusters are a frequent clinical manifestation of PCDH19-related epilepsy, especially in the early stages of the disease. Midazolam infusion has shown marked efficacy. However, a high risk of seizure recurrence and worsening during dose reduction or early withdrawal was reported (60). Intravenous phenytoin and phenobarbital were also used during seizure clusters with good responses (22). The risk of seizure recurrence after ASM withdrawal in PCDH19-related epilepsy is significantly high. A recent study conducted on 42 patients with PCDH19-related epilepsy shows that 88.3% of ASM withdrawal leads to seizure recurrence. In 36.4% of cases, it was also necessary to increase the previous ASM dosage. Only in two cases, it was possible to totally withdraw ASM without seizure recurrence. Suspension of treatment was not only related to a high risk of seizure recurrence (72). Younger age and shorter previous seizure-free periods were risk factors for seizure recurrences, as reported from previous studies conducted on common epilepsies (73). Studies involving a wider population with PCDH19 mutation may improve the management and treatment of this disorder. Greater knowledge of gene variants and pathogenetic mechanisms underlying phenotypic expression could lead to a better understanding of the syndrome and a more effective pharmacological therapy.

**Table 1:** Clinical, EEG and MRI characteristics and treatment efficacy in PCDH19-related epilepsy.

**References**	**Sample size**	**Mean age at seizure onset**	**Seizure semiology**	**EEG patterns**	**Comorbidities**	**MRI findings**	**Treatments**	**Treatments efficacy**
Scheffer et al. (18)	27	14 months	Tonic, tonic-clonic, partial, absence, atonic and myoclonic	Generalized spike wave and polyspike wave, focal discharges with more frequent frontotemporal involvement	ID (15/27) Autistic traits (6/13) Obsessive features (9/27) Aggressive behavior (7/27)	n.a.	VPA, LTG, PHT, PB,	n.a.
Depienne et al. (24)	13	9.5 months	Febrile and afebrile seizure, GTCS, absences, partial and hemiclonic	n.a.	ID (13/13) Behavioral disturbances (5/13) Autistic features (2/13)	n.a.	VPA, CLZ, CLB, TPM, STP, LTG	n.a.
Marini et al. (59)	13	8.5 months	Tonic-clonic, absences, myoclonic and focal	Centroparietooccipital activity (5/13) and frontotemporal activity (2/13)	ID (11/13) Autistic features (5/13)	Normal (13/13)	n.a.	n.a.
Depienne et al. (20)	25	2–54 months	GTCS, tonic, focal, hemiclonic, absence, myoclonic	Normal, focal and generalized seizures	ID (18/25), Behavioral disturbances (7/25)	Normal, frontal median dermoid cyst (1/25)	TPM, LEV, ZNS, CBZ, PB, VPA, LTG, PB, VGB, PHT, STP, CLN, CLB, NTZ	Seizures appeared highly resistant to ASM during the first years of life, the frequency and pharmacoresistance of seizures tended to decrease over time. The only drugs reporting a negative effect were CBZ, LTG and VGB
Marini et al. (19)	35	10 months	Clusters of focal febrile or afebrile seizures. Fearful screaming (24/35)	Prominent involvement of the frontotemporal regions (22/35)	ID (24/35) Autistic traits (11/35)	Normal (35/35)	GVG, OxCZ, LTG, LEV, VPA, PB, TPM, LCM, CZP, ESM, CLB, PHT, CLP, PGB, NZP, DZP	No specific drug or combination of drugs appeared to have been more effective than others. Oral, rectal, or intravenous benzodiazepines had been successful in arresting seizure clusters
Higurashi et al. (60)	18	8.6 months	Tonic, tonic-clonic and focal seizures often with subsequent generalization	Frontal and/or temporal activities (9/18), occipital involvement (4/18)	ID (15/18) Autistic traits (13/18)	Normal (13/18), Frontal heterotopia (1/18), Occipital atrophy (1/18), Hippocampal atrophy (1/18), White matter lesion (1/18)	PHT, BR, CLB, TPM, VPA, CZP, ZNS, PB, CBZ	MDZ showed efficacy in suppressing the ongoing seizure, but was insufficient to manage strong clusters. PHT, BR and CLB were beneficial for decreasing Seizures. CBZ had the poorest efficacy
Harssel et al. (61)	15	4–17 months	Tonic-clonic, tonic, hemiclonic, myoclonic, focal	Focal, multifocal or bilateral synchronous discharges, and background activity was either normal or showed slowing	ID (13/15) Behavioral disturbances (11/15) Autistic trait (6/15)	Normal (14/15), slight asymmetry frontal lobes (1/15)	n.a	n.a
Liu et al. (62)	21	5–18 months	GTCS, focal, myoclonic	Focal or multifocal seizures from the centroparieto-occipital regions or temporal region (5/21). Interictal focal or multifocal epileptic discharges in the centroparietooccipital or frontotemporal regions (14/21)	ID (17/21) Autistic trait (3/21)	Normal (21/21)	PB, LTG, LEV, VPA, TPM, CBZ, TPM, OXC, NZP	Seizures were refractory to antiepileptic drugs at onset in all patients. Seizure frequency and intractability tended to decrease over time
Lotte et al. (63)	58	11.2 months	GTCS (81%)	n.a.	Motor impairment (25/58) ID (48/58) Behavioral disorders (39/58)	Normal (38/58), focal cortical dysplasia (2/58)	BR, CBZ, CLB, CZP, ESM, GBP, CM,LEV, LTG, LZP, NZP, OXC, PB, PER, PGB, PHT, RFN,STM, STP, TPM, VGB, VPA, ZNS	CLB and BR decreased seizure frequency by more than 50% with a responder rate of 68 and 67%, respectively. A long-term response of 50 and 43% respectively was detected after 12 months. PHT resulted particularly ineffective.
Chemaly et al. (56)	13	4–14 months	GTCS, focal, atypical absence	temporo-occipital and frontal onset (8/13)	ID (12/13) Autistic traits (9/13)	Normal (13/13)	VPA,CLZ,VGB, LEV,CLB, PB, STP,TPM, CZP, CBZ, LTG, PHT, LVT, ETX	Clusters responded to benzodiazepines. STP decreased seizure frequency by more than 50%. VGB had a negative impact on behavior in two patients with seizure worsening and was stopped.
Smith et al. (26)	38	11.8 months	Focal, generalized seizures	n.a.	ID (30/38) Behavioral abnormalities (29/38) Autistic features (22/38) Abnormal sleeping patterns (20/25)	Normal	Most frequently used medications include BZP, OXC, VPA, LEV	Uncontrolled seizures with more than 3 medications (23/38), uncontrolled seizures with less than 3 medications (7/38), Controlled seizures with more than 3 medication (5/38), Controlled seizures with less than 3 medication (3/38)
Trivisano et al. (64)	61	10 months	Motor seizures were primarily tonic. Non-motor seizures were characterized by psychomotor arrest, loss of muscle tone, hypopnea, cyanosis and desaturation. Fearful expression was reported as one of the most common initial ictal manifestations.	Interictal epileptiform abnormalities (63.9%). Focal seizures arose from temporal (82.8%), frontal (6.2%), parieto-occipital (6.2%), and central (4.7%) regions. Diffuse onset (39.2%).	ID (36/61). Autistic features (36/61)	Normal	n.a.	During the first decade, epilepsy tended to be active and resistant to multiple antiepileptic drugs. Later on, a decrease in seizures regardless of treatment was observed.
Sadleir et al. (65)	Cohort A: 17 cohort B: 62	Cohort A: n.a. cohort B: 10.3 months	Focal, tonic, GTCS	n.a.	ID (13/17 cohort A) Autistic features (6/17 cohort A)	n.a.	CLB, CBZ, LTG, VPA, TPM, PRD, ACTH, PB, PHT, AZD, DZP, VGB, GP, NZP, TGB, OX, PRD	Levetiracetam resulted in at least 12 months' seizure freedom in 76% of cohort A and in 42% of cohort B

*ACTH, adrenocorticotropic hormone; AZD, acetazolamide; BR, bromide; CBD, cannabidiol; CBZ, carbamazepine; CLB, clobazam; CZP, clonazepam; DZP, diazepam; ESM, ethosuximide; GBP, gabapentin; LCM, lacosamide; LEV, levetiracetam; LTG, lamotrigine; LZP, lorazepam; MDZ, midazolam; NZP, nitrazepam; OXC, oxcarbazepine; PB, phenobarbital; PHT, phenytoin; PER, perampanel; PGB, pregabalin; PHT, phenytoin; PRD, pyridoxine; RFN, rufinamide; STM, sulthiame; STP, stiripentol; TGB, tiagabine; TPM, topiramate; VGB, vigabatrin; VPA, valproate; ZNS, zonisamide*.

## Conclusion

The development of next-generation sequencing techniques has allowed us to bring the diagnosis to a deeper level recognizing specific gene variants and assessing any genotype-phenotype correlations. Besides SCN1A, PCDH19 is among the most relevant genes in epilepsy. The broad phenotypic spectrum of PCDH19-related epilepsy has been extensively studied in recent years and has led to improved and earlier recognition of symptoms. Further studies aimed at a better framing of the non-epileptic features and overall quality of life of these patients are needed. Especially cases of hemizygous males with psychiatric manifestations should be deeper investigated to better understand the expression of this gene and its peculiar inheritance pattern. Most mutations of PCDH19 occur in the extracellular domain, including whole and partial gene deletion and missense, nonsense, and frameshift mutation. The genotype-phenotype correlation has been investigated in several studies; however, it is not yet known how different gene variants can alter clinical manifestations. Protocadherin-19 is known to have an essential role through its extracellular domain in cell adhesion and neuronal architecture (74). However, the role of this molecule is not limited to cell-cell interaction and involves important mechanisms of signal transmission through its intracellular domain. Thus, to optimize precision therapies with the aim of targeting underlying pathogenesis, it is essential to have a greater knowledge of the biological processes in which PCDH19 is involved and how these are altered by the mutations studied so far. In conclusion, it is important to consider PCDH19 in the context of genetic epilepsies. Molecular testing for PCDH19 mutations is recommended especially for female patients who present seizure clusters with early onset, with familiarity or characteristics compatible with GEFS+ or DS, and in cases with cognitive and psychiatric comorbidities.

## Author Contributions

GD and VV put forward the conception of the review and wrote the manuscript. AV and EM participated in the proposal of the concept and revised the manuscript. AF, GT, GD, and PS proposed suggestions for revision. All authors approved the submitted version.

## Conflict of Interest

The authors declare that the research was conducted in the absence of any commercial or financial relationships that could be construed as a potential conflict of interest.

## Publisher's Note

All claims expressed in this article are solely those of the authors and do not necessarily represent those of their affiliated organizations, or those of the publisher, the editors and the reviewers. Any product that may be evaluated in this article, or claim that may be made by its manufacturer, is not guaranteed or endorsed by the publisher.
